# Conserved Glycines Control Disorder and Function in the Cold-Regulated Protein, COR15A

**DOI:** 10.3390/biom9030084

**Published:** 2019-03-02

**Authors:** Oluwakemi T. Sowemimo, Patrick Knox-Brown, Wade Borcherds, Tobias Rindfleisch, Anja Thalhammer, Gary W. Daughdrill

**Affiliations:** 1 Department of Cell Biology, Microbiology, and Molecular Biology, University of South Florida, Tampa, FL 33620, USA; oluwakemi@mail.usf.edu (O.T.S.); wborcher@mail.usf.edu (W.B.); 2 Department of Physical Biochemistry, University of Potsdam, 14476 Potsdam, Germany; knoxbrown@uni-potsdam.de (P.K.-B.); rindflei@uni-potsdam.de (T.R.)

**Keywords:** COR15A, Late embryogenesis abundant, intrinsically disordered proteins, Trifluoroethanol, Nuclear magnetic resonance

## Abstract

Cold-regulated (COR) 15A is an intrinsically disordered protein (IDP) from *Arabidopsis thaliana* important for freezing tolerance. During freezing-induced cellular dehydration, COR15A transitions from a disordered to mostly α-helical structure. We tested whether mutations that increase the helicity of COR15A also increase its protective function. Conserved glycine residues were identified and mutated to alanine. Nuclear magnetic resonance (NMR) spectroscopy was used to identify residue-specific changes in helicity for wildtype (WT) COR15A and the mutants. Circular dichroism (CD) spectroscopy was used to monitor the coil–helix transition in response to increasing concentrations of trifluoroethanol (TFE) and ethylene glycol. The impact of the COR15A mutants on the stability of model membranes during a freeze–thaw cycle was investigated by fluorescence spectroscopy. The results of these experiments showed the mutants had a higher content of α-helical structure and the increased α-helicity improved membrane stabilization during freezing. Comparison of the TFE- and ethylene glycol-induced coil–helix transitions support our conclusion that increasing the transient helicity of COR15A in aqueous solution increases its ability to stabilize membranes during freezing. Altogether, our results suggest the conserved glycine residues are important for maintaining the disordered structure of COR15A but are also compatible with the formation of α-helical structure during freezing induced dehydration.

## 1. Introduction

Intrinsically disordered proteins (IDPs) are proteins that lack a defined three-dimensional structure and exist in various ensembles [1,2]. Intrinsically disordered proteins are found in archaea and bacteria but are most abundant in eukaryotes [3]. The amino acid sequences of IDPs are frequently enriched with repeats of specific amino acid residues or short amino acid motifs, and they rarely form long-range intramolecular interactions [4]. Intrinsically disordered proteins contain a high number of charged and polar amino acid residues, and few hydrophobic residues, which prevents the formation of a hydrophobic core [2,5]. Cold-regulated (COR) 15A is an intrinsically disordered protein from the model plant *Arabidopsis thaliana*, that belongs to the group of late embryogenesis abundant (LEA) proteins. Late embryogenesis abundant proteins accumulate during later stages of seed development and are also found in vegetative tissues of plants [6,7,8]. Late embryogenesis abundant proteins have been previously categorized based on amino acid sequence similarity [8,9]. We are focusing on the archetypical LEA protein COR15A, which is one of the best characterized LEA proteins to date [7,10,11]. During dehydration, either directly administered by modulation of the relative humidity or modelled by increasing solution osmolarity, the intrinsically disordered COR15A accumulates α-helical structure [10,12]. Homology modelling suggests COR15A forms two α-helices connected by a flexible linker in response to water deprivation, that are reversible upon rehydration [10].

Previous work established that overexpression of COR15A which localizes to the chloroplast stroma, increases the tolerance of plant leaves to freezing temperatures and that silencing COR15A and its close homolog COR15B leads to a decrease in freezing tolerance [13]. Late embryogenesis abundant proteins hypothetically function by stabilizing enzymes and membranes [13,14]. In vitro enzyme activity studies have shown that recombinant COR15A stabilizes isolated lactate dehydrogenase by preventing aggregation but has no effect on enzymes that are not prone to aggregation [14,15,16,17,18]. However, in vivo studies suggest that COR15A is not involved in the stabilization of chloroplast-localized enzymes during freezing, but instead stabilizes the chloroplast and plasma membranes [11,14]. Such a membrane-stabilizing function after a freeze–thaw cycle was also reported in vitro, using recombinant COR15A and large unilamellar vesicles (LUVs) modelling the lipid composition of inner chloroplast membrane [14]. Hypothetically, COR15A stabilizes the chloroplast membrane by preventing the lamellar-to-hexagonal phase II transitions as a result of freeze-induced dehydration and by increasing membrane fluidity [14,19]. Interestingly, COR15A interacts with lipids in a partially folded state [12,14,20]. Although COR15A has been studied extensively, there have been no nuclear magnetic resonance experiments performed to examine the atomic level structure and dynamics of COR15A. In this report, the structure and dynamics of COR15A wildtype (WT) and the two mutants predicted to increase helicity were examined in the presence and absence of trifluoroethanol (TFE), and the impact of these mutants on the stability of model membranes after a freeze–thaw cycle was investigated by fluorescence spectroscopy. We further investigated the helix–coil transition of COR15A WT and the mutants in increasing concentrations of TFE and the osmolyte ethylene glycol (EG). TFE is an alcohol based co-solvent that induces/stabilizes helical structure in peptides [21]. If the amino acid sequence of COR15A evolved to be disordered under hydrating conditions and becomes helical under dehydrating conditions, there should be conserved residues that control this behavior. We demonstrate that the α-helicity of COR15A can be increased as a result of specific amino acid substitutions, and this increased α-helicity positively affected its membrane stabilizing function during freezing.

Sequence alignments of various COR15A homologs and AGADIR predictions were used to determine amino acid residues that were likely to impact the α-helical folding state and capacity of COR15A [22,23]. Heteronuclear single quantum coherence (^1^H-^15^N HSQC), HNCACB, HNCO and CBCACONH NMR experiments were used to examine structural differences between COR15A WT and the mutants in the absence and presence of TFE [1,24,25].

## 2. Materials and Methods

### 2.1. Sequence Selection and Alignment

A BLAST search was performed using the non-redundant protein sequences(nr) database with mature COR15A excluding the N-terminal chloroplast localization signal. The alignments were carried out with the Geneious software version 10.0.8 (Biomatters Ltd.) [26] using the Geneious aligner algorithm set to use the Blosum62 matrix with gap open penalty of 12 and extension penalty of 3, and 2 refinement iterations [26]. The accession numbers of the protein sequences are NP_181782, AY587559.1, JF718274.1, EF532304.1, EF526218.1, EU285582.1, FJ594771.1. The sequence alignments of various COR15A homologs were used to determine amino acid residues that were likely to impact the α-helical folding state and capacity of COR15A.

### 2.2. Plasmids and Cell Lines

The codon optimized *cor15a* gene (Invitrogen, Thermofisher Scientific, Carlsbard, CA, USA) was cloned either into a pProEX HTb plasmid or a pET-28a plasmid, which carry a TEV or thrombin cleavage sequence between 6xHIS tag and inserted gene, respectively. Both encode the WT protein sequence. MAAKGDGNILDDLNEATKKASDFVTDKTKEALADGEKAKDYVVEKNSETADTLGKEAEKAAAYVEEKGKEAANKAAEFAEGKAGEAKDATK. Site directed mutagenesis was performed on the wildtype construct without the chloroplast localization signal to obtain the single mutant G68A using the QuikChange Site-Directed Mutagenesis kit (Agilent, Savage, MD, USA). The gene encoding the 4GtoA mutant was synthesized by Invitrogen, Thermofisher Scientific, then sub-cloned into a pET-28a vector and transformed into BL21-DE3 cells from *E. coli* for protein expression. The correct full-length peptide has 140 amino acids of which the signal peptide constitutes the N-terminal 49 amino acids. Thus, the mature COR15A without signal peptide after cleavage of the 6xHIS-tag consists of 91 + 2 amino acids. The latter two are relics from the cleavage sequence between 6xHIS-tag and COR15A sequence.

### 2.3. Protein Production and Purification

#### 2.3.1. Protein Production and Purification for Nuclear Magnetic Resonance Experiments

Uniformly ^15^N-labeled and ^15^N- and ^13^C-labeled samples of COR15A WT and mutants (residues 1–91) were expressed in BL21-DE3 cells grown in M9 medium. They were incubated at 37 °C for about 3 h, then transferred to a 15 °C incubator for 15 min and induced at an OD_600_ of 0.6 with 1 mM isopropyl β-d-1-thiogalactopyranoside (IPTG) and left to grow for 24 h, after which they were centrifuged at 11,280 g for 5 min at 4 °C and pellets were frozen at −80 °C. The pellets were resuspended in nickel load buffer (50 mM NaH_2_PO_4_, 300 mM NaCl, 10 mM imidazole, 0.02% sodium azide, pH 8.0) and lysed with a French press pressure cell using a minimum pressure of 20,000 psi. The lysate was centrifuged at 38,720 g for 1 h. The supernatant was loaded onto a column containing Nickel-NTA resin. The column was washed with nickel load buffer then eluted with nickel elution buffer (50 mM NaH_2_PO_4r_, 300 mM NaCl, 250 mM imidazole, 0.02% sodium azide, pH 8.0). Fractions containing the fusion protein were confirmed using sodium dodecyl sulfate polyacrylamide gel electrophoresis (SDS-PAGE) and dialyzed into gel filtration buffer (50 mM NaH_2_PO_4_, 300 mM NaCl, 1 mM EDTA, 0.02% NaN_3_, pH 7.0) using dialysis tubing with a cutoff of 3.5 kDa. The histidine tag was cleaved using Thrombin CleanCleave Kit (Sigma-Aldrich, St. Louis, MO, USA. The samples were then loaded onto a GE HiLoad 16/60 Superdex 75 column. The column was equilibrated, and the protein eluted with gel filtration buffer at a flow rate of 1 ml/min. Protein purity was verified using SDS-PAGE analysis after the size exclusion.

#### 2.3.2. Protein Production and Purification for Fluorescence Spectroscopy and Circular Dichroism Experiments

The gene containing pProEX Htb vectors were expressed in *E. coli* BL21(DE3) growing in LB medium for 8 h at 37 °C. After cell harvest, cells were resuspended in solubilization buffer (20 mM NaH_2_PO_4_ pH 7.0, 500 mM NaCl), lysed by sonication (Bandelin, SONOPULS) or French press and incubated at 100 °C in a water bath for 10 min. After centrifugation, the supernatant containing heat soluble proteins was purified by affinity chromatography using a Ni^2+^-NTA Sepharose 6 Fast Flow (GE Healthcare, Little Chalfont, UK). COR15A was eluted from the column with solubilization buffer containing 250 mM imidazole. The N-terminal histidine tag was removed by a custom-made TEV protease, due to a TEV-cleavage site within this vector, during overnight dialysis against 10 mM Tris/HCl, pH 8.0; 150 mM NaCl; 1 mM DTT; 0.1 mM EDTA at 4 °C (MWCO 3.5 kDa, Spectrum labs, Los Angeles, CA, USA). Purification was finalized by a second affinity chromatography and a subsequent size exclusion chromatography using a Sepharose 75 26/60 column attached to an ÄKTA system (GE Healthcare, Little Chalfont, UK). Protein solutions were dialyzed against 10 mM TES, 50 mM NaCl and 0.1 mM EDTA, pH 7.4 at 4 C (MWCO 3.5 kDa, Spectrum labs, Los Angeles, CA, USA) or 10 mM NaH_2_PO_4_ and concentrated using an Amicon ultrafiltration cell (Merck Millipore, Darmstadt, Germany) with an MWCO of 3.5 kDa. Protein purity was evaluated by SDS-PAGE and dynamic light scattering. Protein identity was checked by Western Blot analysis using an anti-(His)6 epitope-tag antibody (Dianova GmbH, Hamburg, Germany) and was visualized by an alkaline phosphatase reaction by a secondary antibody (Sigma-Aldrich, Taufenkirchen, Germany).

### 2.4. Nuclear Magnetic Resonance Spectroscopy

The concentration of the WT and mutant proteins used for the NMR experiments were 400–500 μM. Protein concentration was measured using an ND1000 nanodrop. The extinction coefficient of COR15A is 2980 M^−1^ cm^−1^, so we tend to use the 280 nm absorbance data from more concentrated samples to estimate concentration. The lower detection limit of the Nanodrop U/V spectrophotometer used is 0.03 AU. For the NMR experiments, we usually work in the range of 100 μM and above, which gives an absorbance value of about 0.15AU, which is well above the sensitivity limit of the detector. All the protein samples used are diluted from concentrated protein stock solutions, and this method of measuring concentration is used consistently in all three proteins. The reliability of the COR15A protein concentrations calculated using UV absorbance was confirmed using BCA. The NMR experiments on the samples were carried out at 25 °C on the Varian VNMRS 800 MHz spectrometer equipped with a triple resonance pulse field Z-axis gradient cold probe. To make the amide ^1^H and ^15^N as well as ^13^C_α_ and ^13^C_β_ resonance assignments, sensitivity enhanced ^1^H-^15^N HSQC and three-dimensional HNCACB and HNCO experiments were performed on the uniformly ^15^N-labeled and ^15^N- and ^13^C-labeled samples of COR15AWT and mutants in 90% H_2_O, 10% D_2_O, 50 mM NaCl, NaH_2_PO_4_, 50 mM NaCl, 1 mM EDTA, 0.02% NaN_3_, pH 6.8 for the samples without TFE. The samples with TFE were in 70% H_2_O, 20%TFE, 10% D_2_O, 50 mM NaCl, NaH_2_PO_4_, 50 mM NaCl, 1 mM EDTA, 0.02% NaN_3_, pH 6.8. For the HNCACB and HNCO experiment, data were acquired in ^1^H, ^13^C, and ^15^N dimensions using 9689.9228 (t_3_) X 14075.1787 (t_2_) X 1944.3904 (t_1_) H_z_ sweep widths, and 1024 (t_3_) X 128 (t_2_) X 32 (t_1_), respectively. For the HSQC experiments, the sweep width 9689.9228 (t_2_) X 1944.3524 (t_1_), and the increments were 1024 (t_2_) and 128 (t_1_). The NMR spectra were undertaken with a NMRFx Processor and analyzed using NMRViewJ (One Moon Scientific, Inc., Westfield, NJ, USA).

The data from the 2D and 3D NMR experiments were analyzed using the neighbor-corrected intrinsically disordered protein (NCIDP) random coil values and the Vendruscolo δ2D software [24,25]. The random coil values were included in the calculation of the alpha carbon secondary chemical shifts, while the δ2D software was used for the calculation of the % helix values [24,25]. 

### 2.5. Circular Dichroism Spectroscopy

Circular dichroism (CD) measurements were performed in a Jasco J-815 spectrometer (Jasco, Japan) equipped with a thermostatted, Peltier-controlled cell holder. Four spectra were recorded and averaged using quartz cuvettes with a path length of 1 mm (Hellma, Germany) at protein concentrations of 0.10 g/L in 10 mM NaH_2_PO_4_ pH 7.4 in the absence of co-solvent and with increasing concentrations of ethylene glycol (EG) or trifluoroethanol (TFE). All spectra were corrected for buffer contributions and converted to mean residue ellipticities [θ_MRW_] using mean residue weights of 104.1 g/mol, 104.3 g/mol and 104.8 g/mol for COR15A WT, G68A and 4GtoA, respectively. Instrument calibration was done with 1S-(+)-10-camphorsulphonic acid. The ratio of α-helix was estimated using θ_MRW_ at 222 nm [27].

### 2.6. Carboxy Fluorescein (CF) Leakage Assay

All lipids were purchased from Avanti Polar Lipids (Alabaster, AL, USA) and dissolved in chloroform prior to mixing in the respective ratio to model the lipid composition of inner chloroplast membranes (40% monogalactosyldiacylglycerol; 30% digalactosyldiacylglycerol; 15% sulfoquinovosyldiacylglycerol; 15% egg phosphatidylglycerol) referred to as ICMM [14]. A total of 10 mg lipids was dried in a glass tube under a stream of N_2_ at 60 °C and subsequently under vacuum overnight to remove the solvent completely. Dry lipids were rehydrated in 100 mM carboxy fluorescein (CF); 10 mM TES, 50 mM NaCl and 0.1 mM EDTA, pH 7.4 as described previously [28,29]. The mixture was vortexed for 5 s and resuspended multiple times over an interval of 15 min to resolve all the lipids, followed by extrusion through two layers of polycarbonate membranes with 100 nm pore size in a handheld extruder (Avanti Polar Lipids, Alabaster, AL, USA) for the formation of large unilamellar vesicles (LUVs). Liposomes were loaded onto a S75 13/300 size exclusion column connected to the Fast protein liquid chromatography (FPLC) ÄKTA system (GE Healthcare, Freiburg, Germany) to remove free CF. Fractions containing liposomes were detected at 280 nm using the absorption of CF in the ultraviolet (UV) region. The hydrodynamic radius of the liposomes was measured by dynamic light scattering at a scattering angle of 90° with a custom-built apparatus, equipped with a 0.5 W diode-pumped continuous-wave laser (Cobolt Samba 532 nm, Cobolt AB, Solna, Sweden), a high quantum yield avalanche photo diode and an ALV 7002/USB 25 correlator (ALV-GmbH, Langen, Germany) at 23 °C. Hydrodynamic radii of liposomes were calculated from fits of the accumulated autocorrelation functions using the CONTIN algorithm implemented in a custom-made MatLab script (The Math-Works, Natick, MA, USA) [30].

Carboxy Fluorescein containing ICMM LUVs were mixed in equal volumes of respective protein solutions in final molar protein to lipid ratios ranging from 1:50 to 1:200 in polymerase chain reaction (PCR) tubes. Prior to this, protein concentrations were determined by ultraviolet/visible (UV/VIS) spectroscopy using the sequence-specific extinction coefficient at 280 nm of 2560 M^−1^ cm^−1^ valid for all three proteins [31]. Samples were rapidly frozen in an ethylene glycol bath precooled to −20 °C for 2 h [32]. The frozen samples were thawed at 23 °C and transferred to 96 well fluorescent plates. CF leakage was determined with a VIROSKAN FLASH plate reader (Thermo Scientific, Waltham, MA, USA) using an excitation wavelength of 492 nm and an emission wavelength of 517 nm before and after disrupting the liposomes with Triton X-100 (Merck, Darmstadt, Germany). CF leakage from the liposomes was calculated as described previously and normalized to control ICMM LUVs which had not been subjected to a freeze–thaw cycle set as 0% leakage [33]. All proteins were tested for a significance level of *p* < 0.001 compared to ICMM LUVs without protein (w/o) or to COR15A WT, respectively in a one-way analysis of variance (ANOVA).

## 3. Results and Discussion

### 3.1. Sequence Alignments of COR15A

**Figure 1:**
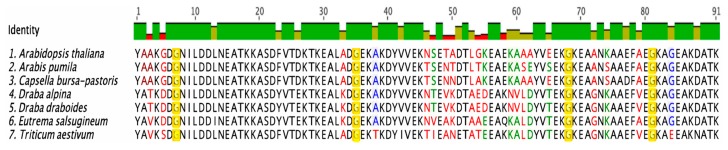
Sequence alignment of cold-regulated (COR) 15A homologs in various plant species. To examine the structure of the *Arabidopsis thaliana* COR15A sequence, alignments were performed using sequences of various species of plants that express this protein. The black letters indicate 100% sequence similarity, the blue letters indicate 80–99%, the green letters indicate 60–79%, and the red letters indicate less than 60% similarity. The residues highlighted in yellow are the glycine residues that are conserved in the examined homologs.

To identify COR15A homologs, a BLAST search using the non-redundant sequence database was performed [34]. Seven COR15A homologs were identified. The sequence alignments were then performed using Geneious and the sequence similarity of the homologs is shown in the multiple protein sequence alignment (Figure 1). One feature that stood out in the alignment was the presence of several highly conserved glycine residues. We thought the conserved glycines were interesting because they are not frequently found in α-helices [35].

The *Arabidopsis thaliana* COR15A protein sequence has 7 glycine residues distributed at the N- and C- termini. Using sequence alignments, the four glycine residues that are conserved in the COR15A sequence across all seven plant species were identified (Figure 1). Glycine residues as structure breakers are known to be predominant in disordered proteins, thus presenting a first indication that the glycine residues are important in regulating the disordered character of COR15A [3,4]. To assess the potential effect of mutating each glycine residue on the structure of COR15A in *Arabidopsis thaliana*, % helicity predictions were performed using AGADIR [22,23].

### 3.2. Structural Characterization of COR15A

**Figure 2:**
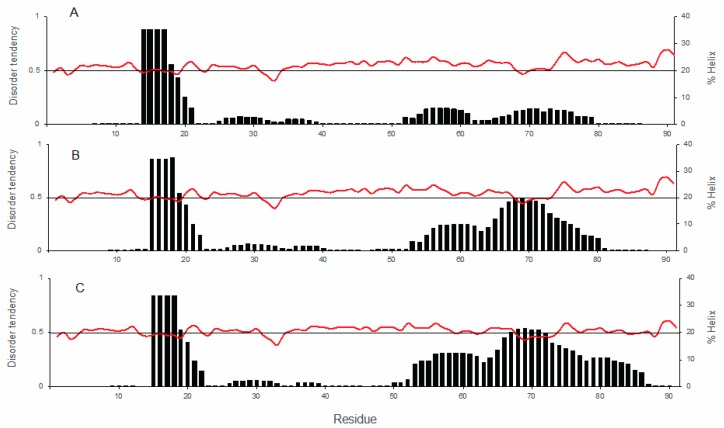
AGADIR % helix and IUPred disorder predictions for individual residues in COR15A wildtype (WT) and mutants. The AGADIR predictions are represented by the black bars, while the IUPred predictions are represented by the red line. (**A**) COR15A WT, (**B**) single mutant G68A, (**C**) quadruple mutant 4GtoA. The primary vertical axis represents disorder tendency as predicted by IUPred, while the secondary vertical axis shows the % helix as predicted by AGADIR. The horizontal axis represents the residue number/position.

Using AGADIR, each of the seven glycine residues in the *Arabidopsis thaliana* COR15A WT sequence was substituted with an alanine residue to test how this affected the % helix prediction in the wildtype protein (data not shown) [22,23]. The glycine residues were substituted one at a time, then multiple glycine exchanges were also tested. Based on the sequence alignments and AGADIR predictions, we designed alanine substitutions at position 68 (G68A) and positions 54, 68, 81, and 84 (4GtoA), because they gave the highest predicted increase in helical content compared to WT (Figure 2). Even though glycines at positions 54 and 84 were not very conserved we mutated them because of the predicted increase in helicity. We analyzed a number of other sequences using AGADIR. The % helicity predictions for five of the six of the COR15A homologs were lower than *Arabidopsis thaliana*. We also looked at sequences that were expected to increase the number of salt bridges which are thought to be important for stabilizing the dehydrated structure of COR15A [10]. None of the salt bridge mutants increased % helicity prediction.

**Figure 3:**
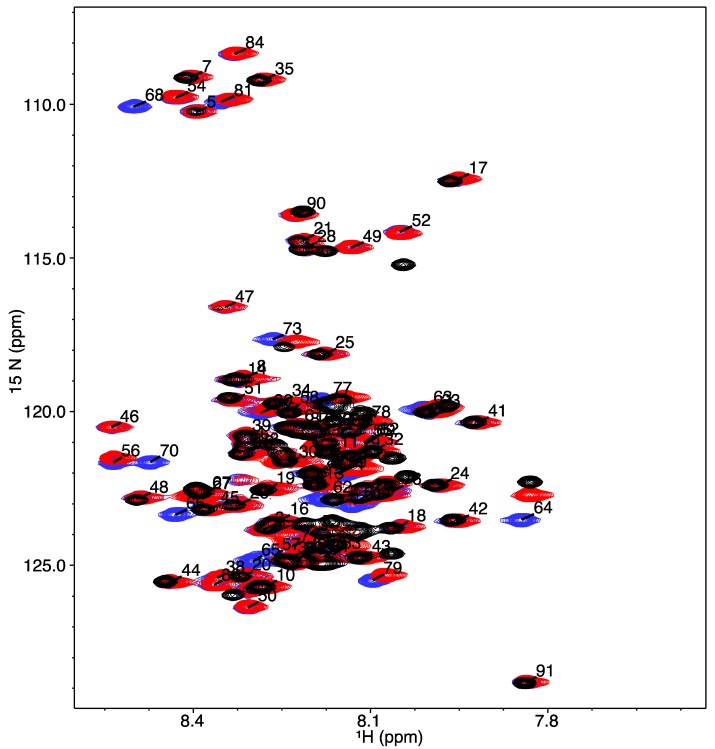
^1^H-^15^N heteronuclear single quantum coherence nuclear magnetic resonance (HSQC NMR) spectra for COR15A WT and mutants in the absence of trifluoroethanol (TFE). The blue peaks depict the WT protein, the red peaks indicate the single mutant G68A and the quadruple 4GtoA mutant is represented by the black peaks. The peaks are labelled with the residue-specific assignments for the WT protein.

**Figure 4:**
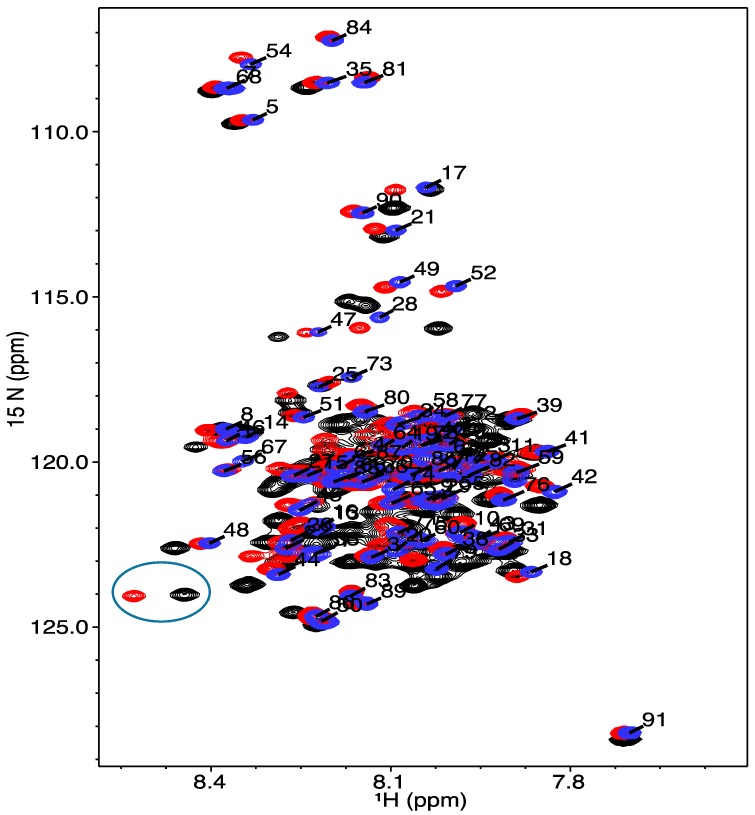
^1^H-^15^N HSQC NMR spectra for COR15A WT and mutants in the presence of 20% TFE. The blue peaks depict the WT protein, the red peaks indicate the single mutant G68A and the quadruple 4GtoA mutant is represented by the black peaks. The peaks are labelled with the residue-specific assignments for the WT protein and the two circled unlabeled peaks at the bottom left represent the G68A residue in the single and quadruple mutant.

AGADIR predicted the presence of two α-helical segments in all three protein sequences, with one segment located in the N-terminal half and the other in the C-terminal half, connected by a linker region (Figure 2A–C). The predicted helicity for the segment closer to the N-terminus is about the same in all three proteins with an average α-helicity of 24% from residues 1–22 (Figure 2A–C). The predicted average α-helicity in the C-terminal segment, residues 53–79, increases from 4% in WT to 11% in the single mutant and 14% in the quadruple mutant. In the IUPred disorder prediction, values above 0.5 predict disorder [36,37]. In Figure 2A–C, the pattern of disorder predicted for the WT and mutant proteins is similar, but there is a reduction in predicted disorder going from the WT (Figure 2A) via G68A (Figure 2B) to 4GtoA (Figure 2C) in the C-terminal half of the sequence. The % α-helix prediction for COR15A WT is in line with the well-known disordered character of the protein in the hydrated state. In Figure 2A–C, it can also be observed that the IUPred disorder prediction for COR15A does not stray far from 0.5, which is an indication that COR15A is on the verge of disorder over the full length of the sequence. Based on our analysis of the AGADIR and IUPred predictions, NMR-labelled recombinant COR15A WT and mutant proteins were expressed and purified [38].

We performed ^1^H-^15^N (HSQC) heteronuclear single quantum coherence NMR experiments for COR15A WT and the two mutants in the absence of TFE (Figure 3). The spectra for all three ^1^H-^15^N HSQC experiments were overlaid using NMRViewJ in order to compare the chemical shift distribution of each residue in the proton and nitrogen dimensions. The distribution of chemical shifts in the nitrogen dimension is dependent on the specific amino acid. In these spectra, it does not indicate any significant structural changes, but we observe disappearance of resonances in the specific regions where glycines are detected (~108 ppm), and the appearance of resonances in the region of the spectra where alanines are detected (~123 ppm) [3].

We previously reported that COR15A folds into predominantly a α-helical structure in response to dehydration and desiccation, and α-helical structure can be induced by either full desiccation or the addition of 20% TFE [10,12,39]. We also performed ^1^H-^15^N HSQC experiments using osmolytes like ethylene glycol and the cosolvent TFE. Analysis of the HSQC spectra of COR15A in 10 M ethylene glycol showed resonance broadening and associated intensity loss (data not shown) due to the effect of increasing viscosity on rotational tumbling of COR15A. In contrast, resonance line shapes and intensities for COR15A in the presence of up to 20% TFE were in a good range for NMR.

The ^1^H-^15^N HSQC spectra of COR15A WT and mutants were overlaid using NMRViewJ, to compare the residue-specific chemical shift dispersion in 20% TFE (Figure 4) as was previously done in the absence of TFE (Figure 3). The distribution pattern of the chemical shifts in the ^15^N and ^1^H dimension indicates structural changes in the backbone structure of the protein, which was not observed in the absence of TFE. This is most apparent in the G68A mutation in the single and the quadruple mutant, which display the largest peak shifts (Figure 4).

**Figure 5:**
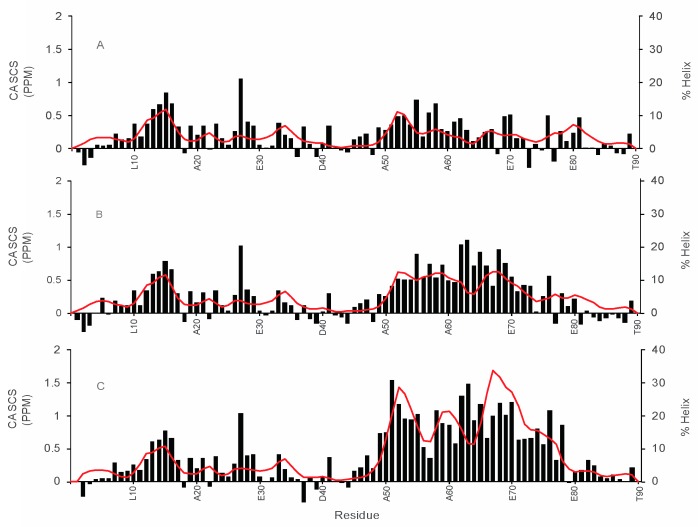
Residue-specific alpha carbon secondary chemical shift and % helix plot for COR15A WT and mutants in 0% TFE. Alpha carbon secondary chemical shifts (black bars) and % helix values (red line) measured using NMR experiments for COR15A WT and mutants in the absence of TFE are shown. The NMR experiments used include ^1^H-^15^N HSQC, HNCACB and HNCO. The alpha carbon secondary chemical shifts are on the primary vertical axis and the measured % helix values are on the secondary vertical axis, while the residue number/position for every 10 residues is on the horizontal axis. (**A**): COR15A WT, (**B**): single mutant G68A, which has the glycine at residue 68 mutated to an alanine, (**C**): quadruple mutant 4GtoA, with glycine residues at positions 54, 68, 81 and 84 mutated to alanine residues.

**Figure 6:**
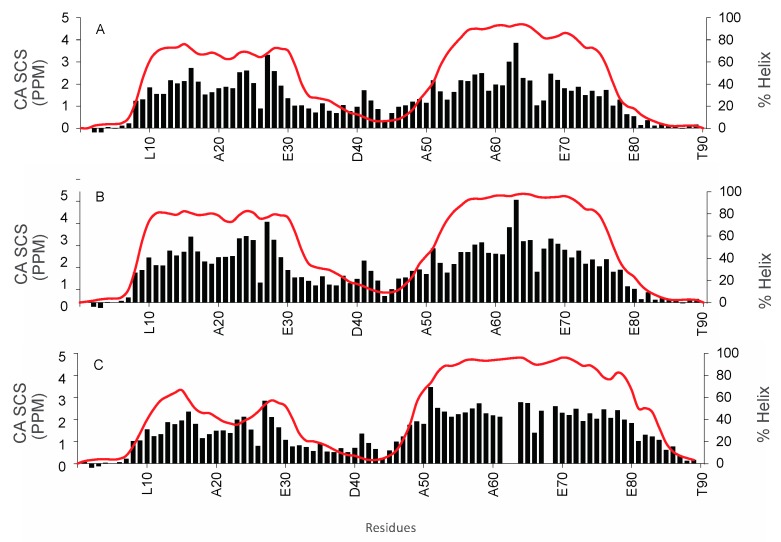
Residue-specific alpha carbon secondary chemical shift and % helix plot for COR15A WT and mutants in the presence of 20% TFE. Alpha carbon secondary chemical shifts (black bars) and % helix values (red line) measured using δ2D data for COR15A WT and mutants in the presence of 20% TFE. The alpha carbon secondary chemical shifts are on the primary vertical axis and the measured % helix values are on the secondary vertical axis, while the residue number/position for every 10 residues is on the horizontal axis. (**A**): COR15A WT, (**B**): single glycine mutant G68A, (**C**): quadruple mutant 4GtoA, with glycine residues at positions 54, 68, 81 and 84 mutated to alanine residues.

To further characterize the structural changes that may be occurring in the WT and mutant proteins, backbone resonance assignments were made using data from ^1^H-^15^N HSQC, HNCACB, HNCO and CBCACONH experiments. Using the resonance assignments, the alpha carbon secondary chemical shifts and residue specific % helicity values were calculated and plotted (Figure 5 and Figure 6).

Figure 5 shows the alpha carbon secondary chemical shifts and the % helix values calculated from all the backbone NMR chemical shifts using δ2D, in 0% TFE, for COR15A WT and the G68A and 4GtoA mutants [24]. % helix, as labeled on the y-axis of Figure 5 and Figure 6 is equal to the δ2D values multiplied by 100. The plots (Figure 5) indicate that the first 10 residues from the N- and C- termini of all three proteins have very little α-helical structure. The average α-helical content of the N-terminal α-helix (residues 10–31) is 4.6% in all three proteins, which is about a quarter of the % α-helix value predicted by AGADIR. The C- terminal α-helix, ranging from residues 50–84, increases from 4.6% in WT to 7.9% in G68A to 16.4% in 4GtoA. The two mutants show increased α-helicity, which is restricted to the C-terminal segment (Figure 5A–C). The overall α-helical content in 0% TFE of the WT protein (3.7%) is similar to that calculated using the Chen algorithm (3.5%), but less than that obtained from CDpro (5.5%), using data from previously performed circular dichroism experiments [10,27,40]. Furthermore, the overall average α-helical content of the three proteins as calculated using NMR data are less than the values predicted using AGADIR. The values calculated from the NMR data for WT is 3.7%, the single mutant is 5% and the quadruple mutant is 8.3%, while those calculated from the AGADIR predictions are 4.4% WT, 6.8% G68A and 8% 4GtoA. The α-carbon secondary chemical shifts for the three proteins at the N-terminus are about the same, and it is approximately 0.33 ppm, but at the C-terminus, it increases from 0.3 ppm in WT to 0.5 ppm in G68A to 0.8 ppm in 4GtoA. The increase in the α-carbon secondary chemical shifts in the N-terminal segments of the three proteins indicates an increase in α-helical content from WT to the single and quadruple mutants. The analysis of the secondary chemical shift values for COR15A WT and mutants was also performed for the 20% TFE samples to compare the changes in the α-helical content of the proteins in the hydrated state (Figure 5A–C) and the dehydrated state which is mimicked by the addition of 20% TFE (Figure 6A–C) [21,25].

In 20% TFE, both α-helical segments in the N- and C-terminal half of COR15A WT and mutants are altered. There is a significant increase in α-helical content compared to the samples without TFE (Figure 5A–C). The middle region is proposed to maintain its disordered nature even in the presence of TFE, thus acting as an unstructured linker and the α-helical population of the residues present is similarly low in all three proteins (Figure 6A–C). The N-terminal α-helical population increases from 67.6% in WT (Figure 6A) to 77.6% in the single mutant (Figure 6B). However, in the quadruple mutant 4GtoA, the α-helical content of the N-terminal segment is decreased to 48.1% (Figure 6C). This decrease in the α-helical population in the N- terminal segment (residues 10–31) is also apparent in the α-carbon secondary chemical shifts, where the average WT value is 1.97 ppm, G68A is 2.19 ppm and 4GtoA is 1.63 ppm, even though there are no mutations in the N-terminal segment. The C-terminal α-helical segment from residues 50–84 has a higher average of α-helical content compared to the N-terminal segment in all three proteins, and the 4GtoA mutant also has the lowest values in the N-terminal segment. Additionally, the C-terminal α-helix is extended towards the C-terminus exclusively in the 4GtoA mutant, which harbors all 4 glycine to alanine mutations in the C-terminal segment (Figure 6C), compared to the single mutant with one mutation in the C-terminal segment and the WT (Figure 6A,B). The C-terminal α-helix, which includes residues 50–84, increases from 65.2% in WT to 72.3% in the single mutant to 82.8% in the quadruple mutant. The overall α-helical content of the WT protein in 20% TFE from the NMR experiments (~47.5%) is similar to that calculated using the Chen algorithm (49.7%), but less than that obtained from CDpro (56.4%), using data from previously performed circular dichroism experiments [10,27,40]. The α-carbon secondary chemical shifts at the C-terminus, increases from 1.66 ppm in WT to 1.95 ppm in G68A to 2.19 ppm in 4GtoA. The increase in the α-carbon secondary chemical shifts in the C-terminal segments of the three proteins indicates an increase in α-helical content from WT to the single and quadruple mutants.

Since we could not collect high-quality NMR spectra of COR15A in ethylene glycol, we used CD to confirm the levels of helicity induced by TFE were consistent with those induced by ethylene glycol, which is important since we argue that the TFE-induced structure may be similar to what is observed during freezing induced dehydration.

**Figure 7:**
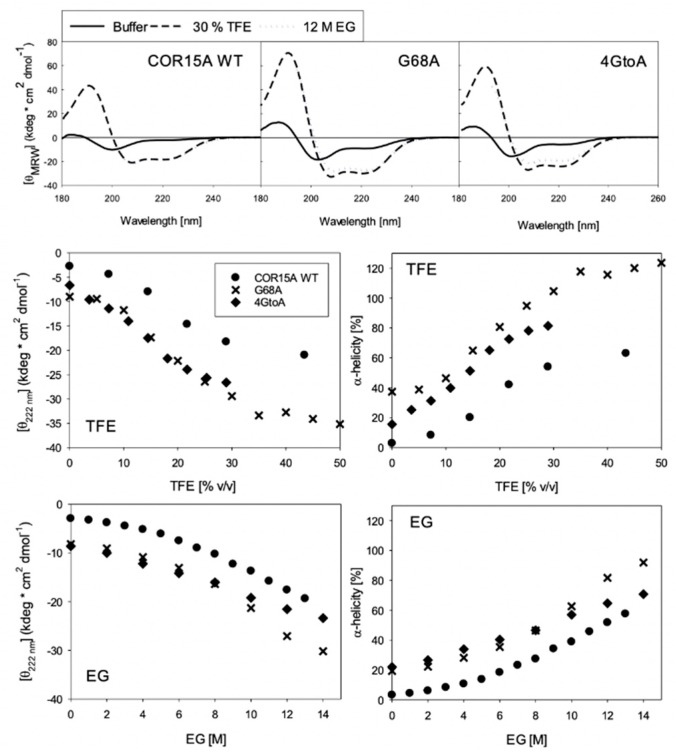
Far-ultraviolet (UV) circular dichroism (CD) spectra in buffer, 20 (*v*/*v*) % TFE and 12 M Ethylene Glycol for COR15A WT, G68A and 4GtoA (**A**). The mutants have more α-helical spectra than the WT in buffer alone, and in the presence of high concentrations of both co-solvents. Coil-helix transitions of COR15A WT and both mutants in TFE (**B**) and EG (**C**), specified by θ_MRW_ at 222 nm (left panels) and derived α-helix ratios (right panels).

Far-UV CD spectroscopy was used to investigate secondary structure transitions of COR15A WT and mutants in response to increasing concentrations of the co-solvents TFE and ethylene glycol (EG) (Figure 7). Both mutants are more α-helical in buffer and in high concentrations of TFE, in agreement with the NMR data. The osmolyte EG presents a useful model system for the severely reduced water availability in a cellular environment during freezing, which COR15A encounters under physiological conditions. The concentration range of EG used in our analysis corresponds to osmolarities plant cells encounter in physiological freezing temperatures down to about −30 °C [41]. Interestingly, all three proteins show comparable CD spectra in 30% TFE and 12 M EG, indicating that a similar coil–helix transition of COR15A and the mutants can be induced by both co-solvents and thus underlining the relevance of the NMR data acquired in TFE. The coil–helix transitions seem to be mostly complete at 30% TFE for COR15A WT and G68A. We were not able to record CD spectra of 4GtoA in sufficiently high TFE concentrations to reach a stable post-transition stage due to protein aggregation, so we cannot state a similar finding for 4GtoA. It is obvious from the CD spectra and the derived α-helicity that the latter must be slightly overrated, which is most likely due to an underestimation of protein concentration. This is a general problem for IDPs due to the underrepresentation of aromatic amino acids [42]. Thus, the α-helix ratios cannot be directly compared to those derived from NMR analyses. However, as all three proteins present an identical molar extinction coefficient at 280 nm, a direct comparison of the ellipticities and the derived α-helicity among the proteins is valid. Both mutants are noticeably more α-helical than the WT in the absence of co-solvent and throughout the complete co-solvent induced transition. As the transitions in most cases lack well-defined pre- and post-transition baselines, the co-solvent concentration in the transition midpoints cannot be determined exactly but only estimated to be similar for all three proteins. Differences between the mutants are obvious. G68A is more α-helical than 4GtoA over the whole range of TFE concentrations and at EG concentrations above 10 M, thus corroborating and strengthening the NMR data in 20% TFE. Interestingly, the α-helicity of 4GtoA and G68A are similar in the absence of co-solvent. However, upon increasing TFE and EG concentrations, G68A becomes considerably more α-helical than 4GtoA, evidencing a higher overall folding propensity. In contrast, the overall folding propensity of 4GtoA is similar to the WT.

Taken together, the structure of COR15A in 20% TFE closely resembles that obtained from homology modelling in a vacuum, both describing a mainly α-helical molecule, made of two α-helical regions connected by a flexible linker [10]. Interestingly, there are distinct differences between the mutants in 20% TFE, when looking at changes in the α-helicity of the N-terminal segment. In the single G68A mutant, the overall α-helicity of the N-terminal segment is increased. In contrast, the mutation of four C-terminal glycine residues reduces the α-helical content of the N-terminal segment. These findings point towards the apparent presence of long-range interactions between the two α-helices, indicating a mutual stabilization. This is apparently increased by the single GtoA mutation and decreased by the mutation of the four glycine residues. Considering the amphipathic nature of the modeled α-helices, such a mutual stabilization is likely achieved by hydrophobic interaction [10]. In such a scenario, the mutated glycine residues may be crucial for a proper orientation of the hydrophobic α-helical segments towards each other, which would be compromised by the GtoA mutations.

### 3.3. Functional Characterization of COR15A

Previous research has indicated that the interaction of COR15A with galactolipids of inner chloroplast membranes may be the mechanism by which COR15A increases *Arabidopsis* freezing tolerance [19]. To test the effectiveness of the COR15A mutants compared to the WT protein in stabilizing these membranes during a freeze-thaw cycle, leakage of the fluorescent dye carboxy fluorescein from LUVs mimicking the lipid composition of these membranes was measured in the presence and absence of recombinant proteins in different protein: lipid molar ratios.

**Figure 8:**
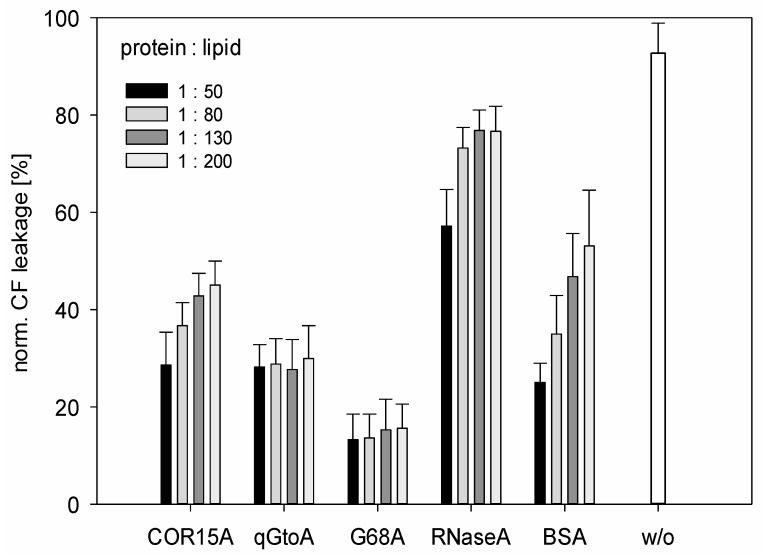
α-helical mutants reduce carboxy fluorescein (CF) leakage from vesicles. Carboxy flourescein leakage of inner chloroplast membranes (ICMM) large unilamellar vesicles (LUVs) after freezing and subsequent thawing was performed using the fluorescence signal detected by λ_ex_ = 492nm and λ_em_ = 517nm. The protein:lipid molar ratios used were 1:50, 1:80, 1:130, 1:200, and these are shown in different shades. All proteins showed a significance level *p* < 0.001 compared to ICMM LUVs without protein (*w*/*o*). Error bars refer to the standard derivation of triplicates from up to three experiments.

Liposomes without any protein added were strongly compromised after freezing and subsequent thawing (Figure 8), with dye leakage of about 90%, which is directly proportional to LUV damage. Similar to previously reported results, COR15A WT significantly stabilized the LUVs in a concentration-dependent manner during a freeze–thaw cycle, with dye leakage between 30–50% [16]. The degree of liposome damage in the presence of the two mutants is less than in the presence of the WT protein. The quadruple mutant 4GtoA showed a small but still significantly better LUV stabilization than COR15A WT (*p* < 0.001) in protein:lipid molar ratios above 1:50. G68A stabilized the LUVs significantly better (*p* < 0.001) than COR15A WT in all tested protein:lipid ratios. This is an interesting finding when combined with the higher overall α-helicity of G68A compared to 4GtoA in high co-solvent concentrations, assuming that COR15A associates with and consequently stabilizes membranes in a folded state as induced by the investigated co-solvents. This finding supports the hypothesis that actually COR15A functionality is directly related to α-helicity. Previous reports suggested that COR15A might form oligomeric structures in the fully hydrated state, as shown by crosslinking experiments [16]. If it actually does so under conditions of reduced water availability, as experienced during freezing, has not been investigated. Thus, we cannot rule out the possibility that COR15A oligomer formation might be impacted in the mutants, thus influencing functionality. In contrast to COR15A WT, the protective effect of the mutants was independent of the lipid:protein ratio, indicating that a lower amount of mutant protein is sufficient to tap their stabilization potential. The reference protein RNase A had a minor, nevertheless significant protective effect on liposome stability, whereas Bovine serum albumin, as a known membrane stabilizer, significantly better protected the LUVs from dye leakage in a concentration dependent manner, compared to COR15A WT. These data corroborate the previous finding of COR15A associating with membranes exclusively in a folded state [12,43]. The folding state seemingly does not only influence membrane association but consequently also membrane stabilization. So, what is the apparent advantage of COR15A in being intrinsically disordered? The coil-helix transition of COR15A is strictly modulated by the osmolarity of the cellular environment, which increases with decreasing freezing temperature [41]. The major finding we report here is that increased α-helicity of COR15A directly translates into increased functionality. This presents crucial progress in understanding the structure–function relationship of COR15A specifically and should be investigated regarding other LEA proteins in the future with respect to the long-term perspective of manufacturing plants with improved desiccation, dehydration or freezing tolerance.

## 4. Conclusions

This study examines the importance of conserved glycine residues on maintaining the disordered structure of COR15A using sequence alignments. The role of increasing helicity on the membrane-stabilizing function of COR15A. The sequence alignments in Figure 1 show that glycine residues at positions 7, 35, 68, and 81 in the COR15A sequence are conserved across several plant species. In addition, the glycine residue at position 84 is mostly conserved and that at position 54 is not. AGADIR predicted that the glycine residue at position 68 had a stronger impact on COR15A disorder than the remaining glycine residues. It also predicted that the glycine residues at positions 54, 68, 81 and 84 in the C-terminal segment of the protein had a stronger impact on the preservation of COR15A disorder compared to the glycine residues in the N-terminal segment. Based on these indications, we made a single glycine to alanine mutation at position 68 and a quadruple mutant at residues 54, 68, 81 and 84. The mutant proteins and COR15A were subject to a detailed structural and functional analysis.

In previous work by Thalhammer and colleagues, a homology model was published for the dehydrated state of COR15A. In this model, COR15A forms a structure with N- and C-terminal helices separated by a long loop [10]. The length and flexibility of the long loop may control interactions between the N- and C-terminal helix. In 0% TFE (Figure 5A–C), the N-terminal helix (residues 10–31) has an average helicity of 4.6% and is not affected by C-terminal mutations. However, the helicity of the C-terminal helix (residues 50–84) increases from WT (4.6%) to G68A (7.9%) to 4GtoA (16.4%). The 20% TFE samples, show a different trend. The helical content of the N-terminal helix increases from WT (67.6%) to G68A (77.6%), but in 4GtoA (48.1%), the helical content decreases. However, in the C-terminal helix, there is an increase in helical content from WT (65.2%) to G68A (72.3%) to 4GtoA (81.6%). Based on this analysis, we think it is possible that the observed decrease in the helical content of 4GtoA in 20% TFE, may be due to a decrease in the interaction of the two helical segments in 20% TFE due to the G54A mutation.

The NMR and CD studies performed on the WT and mutant proteins showed that in the absence of TFE, COR15A WT had lower α-helicity compared to both mutants. As expected, in the presence of the α-helix-inducing agent TFE, α-helicity was increased in all proteins. While both mutants had higher α-helicity than the WT, the single mutant had higher α-helicity than the quadruple mutant in the N-terminal α-helix. Functionally, higher α-helicity resulted in increased liposome stabilization in response to freezing (Figure 8). This effect was greater for the G68A mutant, which showed the highest overall α-helical content in high TFE and EG concentrations. This finding is consistent with our current understanding of membrane association of COR15A via α-helical segments [10,11,14]. The functionality of COR15A is modulated by the folding state of the protein and more helical variants appear to provide greater protection to the membrane. The impact of this observation is significant, considering the possibility of increasing plant responses to dehydrative stress simply by shifting the coil–helix equilibrium towards the more folded conformation.
